# High Stretch Associated with Mechanical Ventilation Promotes Piezo1-Mediated Migration of Airway Smooth Muscle Cells

**DOI:** 10.3390/ijms25031748

**Published:** 2024-02-01

**Authors:** Mingzhi Luo, Rong Gu, Chunhong Wang, Jia Guo, Xiangrong Zhang, Kai Ni, Lei Liu, Yan Pan, Jingjing Li, Linhong Deng

**Affiliations:** Changzhou Key Laboratory of Respiratory Medical Engineering, Institute of Biomedical Engineering and Health Sciences, School of Medical and Health Engineering, Changzhou University, Changzhou 213164, China; s21091055023@smail.cczu.edu.cn (R.G.); s21091055096@smail.cczu.edu.cn (C.W.); guojia@cczu.edu.cn (J.G.); b21010805004@smail.cczu.edu.cn (K.N.); bioll@cczu.edu.cn (L.L.); py@cczu.edu.cn (Y.P.); jingjingli@cczu.edu.cn (J.L.)

**Keywords:** high stretch, airway smooth muscle cells, migration, Piezo1, integrin

## Abstract

Ventilator-induced lung injury (VILI) during mechanical ventilation (MV) has been attributed to airway remodeling involving increased airway smooth muscle cells (ASMCs), but the underlying mechanism is not fully understood. Thus, we aimed to investigate whether MV-associated high stretch (>10% strain) could modulate mechanosensitive Piezo1 expression and thereby alter cell migration of ASMCs as a potential pathway to increased ASMCs in VILI. C57BL/6 mice and ASMCs were subjected to MV at high tidal volume (V_T_, 18 mL/kg, 3 h) and high stretch (13% strain, 0.5 Hz, 72 h), respectively. Subsequently, the mice or cells were evaluated for Piezo1 and integrin mRNA expression by immunohistochemical staining and quantitative PCR (qPCR), and cell migration and adhesion by transwell and cell adhesion assays. Cells were either treated or not with Piezo1 siRNA, Piezo1-eGFP, Piezo1 knockin, Y27632, or blebbistatin to regulate Piezo1 mRNA expression or inhibit Rho-associated kinase (ROCK) signaling prior to migration or adhesion assessment. We found that expression of Piezo1 in in situ lung tissue, mRNA expression of Piezo1 and integrin αVβ1 and cell adhesion of ASMCs isolated from mice with MV were all reduced but the cell migration of primary ASMCs (pASMCs) isolated from mice with MV was greatly enhanced. Similarly, cell line mouse ASMCs (mASMCs) cultured in vitro with high stretch showed that mRNA expression of Piezo1 and integrin αVβ1 and cell adhesion were all reduced but cell migration was greatly enhanced. Interestingly, such effects of MV or high stretch on ASMCs could be either induced or abolished/reversed by down/up-regulation of Piezo1 mRNA expression and inhibition of ROCK signaling. High stretch associated with MV appears to be a mechanical modulator of Piezo1 mRNA expression and can, thus, promote cell migration of ASMCs during therapeutic MV. This may be a novel mechanism of detrimental airway remodeling associated with MV, and, therefore, a potential intervention target to treat VILI.

## 1. Introduction

Mechanical ventilation (MV) is essential in life support for critically ill patients with acute respiratory distress syndrome (ARDS), but it can also cause or aggravate lung damage due to excessively high stretch (>10% strain) loaded on the airways during treatment, a phenomenon called ventilator-induced lung injury (VILI) [1,2,3,4]. Such high stretch has been reported to induce several pathological responses including pulmonary fibrosis (PF), which is characterized by progressive and irreversible pathogenic airway remodeling involving airway smooth muscle cells (ASMCs) [5,6,7,8,9,10]. For example, young children aged 2 weeks to 3.5 years who were born as early as 23–24 weeks preterm and, thus, required MV show an increased amount of ASMCs in the walls of small airways [11,12,13]. This consequence can be explained in part by the well-recognized phenomenon of enhanced cell proliferation of ASMCs in response to cyclic stretch [14,15]. However, few have studied well the phenomenon and underlying mechanism of stretch-modulated migration behavior of ASMCs in relation to VILI.

Of particular interest are the roles of mechanosensors such as integrin and Pieso1 in mediating migration behaviors of ASMCs during high-stretch conditions [16,17,18]. It is well known that cell migration involves integrins because the interaction between integrins and ligands in the extracellular matrix determines the adhesion and, thus, migration speed of the cell [19]. Moreover, cell migration has been characterized by two distinct modes, namely, slow integrin-dependent or fast integrin-independent cell migration, and the transition between these two modes was predominantly determined by the level of cell adhesion and expression of various members of the integrin family, especially integrin αV and β1 in ASMCs [20,21]. For example, reduced expression of integrin α2β1 and weakened cell adhesion are associated with cell migration transition towards the integrin-independent mode in human fibrosarcoma and non-small lung carcinoma cells [22]. Low adhesion and strong confinement can also induce fast integrin-independent migration in several other types of slow mesenchymal tumor cells [23].

The integrin-associated cell adhesion and migration are also known to be mediated by the mechanosensitive channel Piezo1 [24,25,26,27,28]. For example, loss of Piezo1 has been found to enhance integrin-independent migration by decreasing integrin affinity in airway epithelial cells [29]. In contrast, loss of Piezo1 has been found to impair cell migration by decreasing integrin expression in gastric epithelial cells [30]. This contradiction suggests that the function of Piezo1 in the mediation of cell migration may be different depending on not only the cell type but also the extracellular conditions to which the cells are exposed.

Furthermore, integrin expression is modulated by Rho-associated kinase (ROCK) signaling, which acts as an essential controller during integrin-independent migration transition via regulation of myosin activity, molecular reprogramming, and traction force in the cells [31]. For instance, in tumor cells, the integrin α2β1 expression reduced by protease inhibitors can be restored when ROCK signaling is inhibited, which leads to impaired integrin-independent cell migration [22]. On the other hand, recent studies have shown that the ROCK pathway can be activated by high stretch in non-muscle cells involved in various lung pathological conditions including VILI [32,33,34]. These findings from tumor and non-muscle cells suggest that high stretch may activate ROCK signaling to modulate integrin and Piezo1 expression and, thus, eventually induce the cell migration mode transition. In addition to that, we found in more recent studies that stretch could reduce the expression of integrin and Piezo1 in cultured ASMCs [35]. However, it is not clear whether and how such stretch-induced down-regulation of Piezo1 and integrin would impact the cell migration behavior of ASMCs as an underlying mechanism of contribution to airway remodeling in VILI.

Thus, in the present study, we systematically evaluated the effects of high stretch on Piezo1 and integrin αVβ1 mRNA expression and the cell migration/adhesion behavior of ASMCs both in vivo and in vitro, using mouse models under large tidal volume (V_T_) via MV and cell culture models subjected to 13% cyclic strain, respectively. Taken together, our results suggest that Piezo1 modulation during high stretch and the resultant increase in migration of ASMCs may be a new mechanism to contribute to the pathological airway remodeling associated with MV and, thus, provide a potential therapeutic target to mitigate VILI.

## 2. Results

### 2.1. Mechanical Ventilation Decreased Piezo1 mRNA Expression in Lung Tissue In Vivo with Promoted Cell Migration of ASMCS

**Figure 1:**
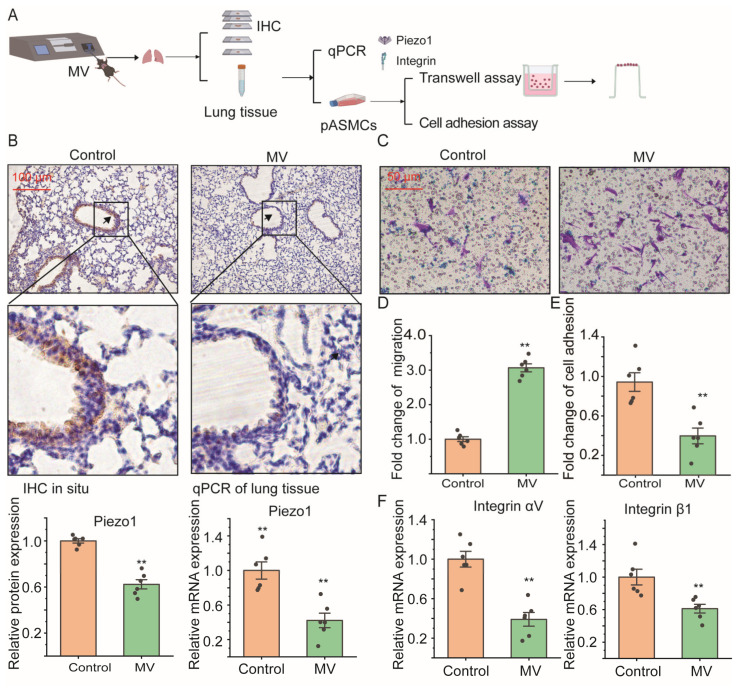
Effect of mechanical ventilation (MV) on Piezo1 and integrin mRNA expression in mouse lung tissue, and on in vitro cell migration and adhesion of mouse primary ASMCs (pASMCs). (**A**) The schematic overview of this experimental design. (**B**) Upper panel: representative immunohistochemistry (IHC) staining images of Piezo1 level in in situ lung tissue of mice under spontaneous breathing (control) or MV with high tidal volume (V_T_, 18 mL/kg, 3 h). Brown and blue color in the image indicate positive staining of Piezo1 and nuclei stained with hematoxylin, respectively. Arrows indicate airway walls. Scale bar = 100 μm. Lower panel: quantitative Piezo1 protein expression (left) by IHC and mRNA expression (right) by quantitative PCR (qPCR) in lung tissue of mice in control vs. MV groups. (**C**,**D**) Representative images and quantification of migration in transwell assay of pASMCs isolated from mice in control vs. MV groups. Scale bar = 50 μm. (**E**) Quantification of cell adhesion by cell adhesion assay of pASMCs isolated from mice in control vs. MV groups. (**F**) Quantification of integrin (αV, β1) mRNA expression by qPCR of lung tissue isolated from mice in control vs. MV groups. In all cases, n = 6. Markers denote significant differences from control conditions in the static group (** *p* < 0.01).

The Piezo1 expression and integrin (αV, β1) mRNA expression of lung tissue were detected in spontaneous-breathing or MV-treated mice with immunohistochemistry (IHC) and quantitative PCR (qPCR); cell migration and cell adhesion were also detected with a transwell assay and cell adhesion assay of isolated primary ASMCs (pASMCs) from these mice, respectively (Figure 1A). In Figure 1B, the upper panel shows representative microscopic images of IHC-stained Piezo1 in lung tissue sections from mice after spontaneous breathing (control) or high-tidal-volume (V_T_, 18 mL/kg, 3 h) mechanical ventilation (MV). Piezo1 expression in in situ lung tissue was abundant in control subjects but much less in MV-treated mice (see arrow-pointed regions and the magnified views). The lower left panel shows quantitative densitometry results of Piezo1 in lung tissue. The results show that Piezo1 expression was reduced by ~40% (*p* = 0.001) in mice under MV as compared to the control. The mRNA expression of Piezo1 in lung tissue quantified by qPCR showed ~60% reduction in lung tissue from MV-treated mice compared with control mice (*p* = 0.001, Figure 1 lower right panel). The pASMCs from the control mice showed abundantly expressed Piezo1 in the cell membrane, plasma, and nucleus in punctate structures, but those from MV-treated mice showed lower expression of Piezo1 (Figure S1A). Figure S1B shows the quantified mean fluorescence intensity of Piezo1, and the results show that high stretch significantly decreased Piezo1 intensity in total cells, cell membrane, cytosol, and nucleus. But the ratios of cytosol/nucleus to cell membrane fluorescence intensity did not change in pASMCs isolated from both static and MV conditions.

Figure 1C shows representative images of cell migration in the transwell assay of pASMCs. It shows clearly that compared to the control group, more cells in the MV group migrated through the pores of the transwell membrane. Figure 1D shows that the cell migration of pASMCs was increased by ~2-fold due to MV as compared to control conditions (*p* = 0.001). In contrast, Figure 1E shows cell adhesion of pASMCs decreased by ~60% (*p* = 0.001) due to MV as compared to the control group. Figure 1F shows that cell adhesion and mRNA expression of the integrin (αV, β1) of lung tissue all decreased by ~60% (*p* = 0.001) and ~40 (*p* = 0.006) due to MV as compared to the control group.

### 2.2. High Stretch Decreased Piezo1 mRNA Expression and Promoted Migration Transition of ASMCs In Vitro

**Figure 2:**
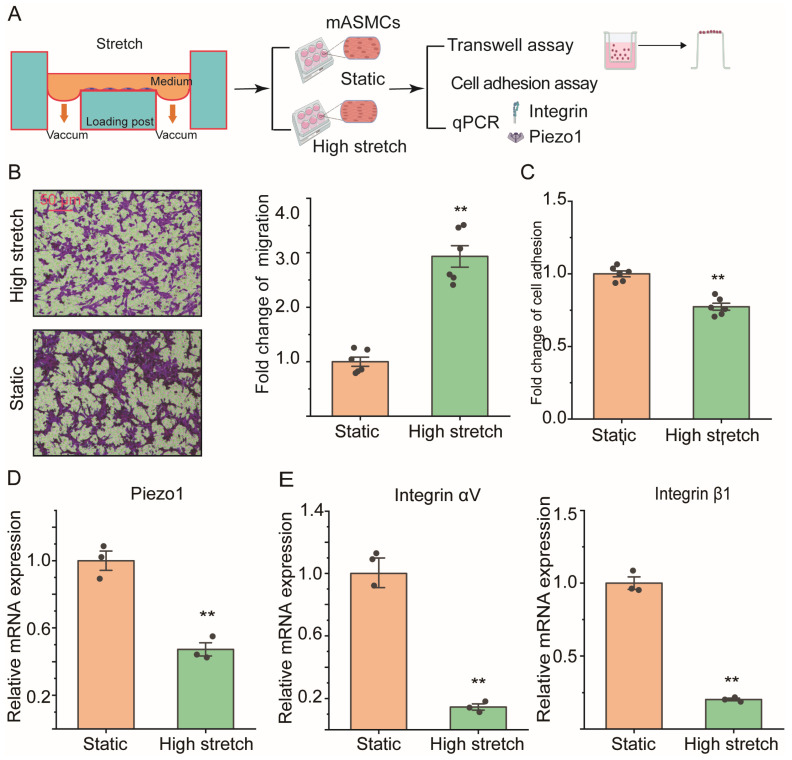
Effect of high stretch on cell migration and adhesion, Piezo1, and integrin mRNA expression of in vitro-cultured cell line mouse ASMCs (mASMCs). (**A**) The schematic overview of this experimental design. (**B**,**C**) Representative images, and quantification of cell migration in transwell assay of mASMCs cultured under either static or high-stretch condition (Static vs. High stretch). Scale bar = 50 μm; n = 6. (**D**,**E**) Quantification of cell adhesion by cell adhesion assay, mRNA expression of Piezo1 and integrin (αV, β1) by qPCR, of mASMCs in static vs. high-stretch groups, n = 3. Markers denote significant differences from the static group (** *p* < 0.01).

Cell migration and adhesion, Piezo1, and integrin mRNA expression of cultured cell line mouse ASMCs (mASMCs) in static or high-stretch conditions were detected with transwell assay, cell adhesion assay, and qPCR, respectively (Figure 2A). Figure 2B (left panel) shows representative images of cell migration in the transwell assay of mASMCs cultured in static or high-stretch conditions (static vs. high stretch). Quantification of cell migration by transwell assay and cell adhesion by cell adhesion assay of mASMCs was shown in Figure 2B (right panel) and Figure 2C, respectively. Similarly to the in vivo effect of MV on mice, high stretch in vitro enhanced cell migration by ~2-fold (*p* = 0.001), and reduced cell adhesion by ~25% of mASMCs as compared to controls (High stretch vs. Static, *p* = 0.001).

Figure 2D,E shows mRNA expression of Piezo1 and integrin in mASMCs cultured in static or high-stretch conditions (static vs. high stretch). Interestingly, high-stretch conditions also reduced mRNA expression of Piezo1 by ~50% (*p* = 0.002) and integrin by ~80% (αV: ~80%, *p* = 0.021, β1: ~60%, *p* = 0.001, respectively) compared to static conditions.

### 2.3. Loss of Piezo1 Promoted Cell Migration but Reduced Cell Adhesion and Integrin mRNA Expression of ASMCs In Vitro

The mASMCs transfected with Piezo1 siRNA, mPiezo1-eGFP showed mRNA expression of Piezo1 reduced by ~75% (*p* = 0.003), and increased by ~23-fold (*p* = 0.001) as compared with Scramble, and Vector, respectively, verifying the successful down/up-regulation of Piezo1 mRNA in the cells (Figure S2A). Results of cell counting kit 8 (CCK-8) (*p* = 0.819 and 0.135 for Piezo1 down/up-regulation, respectively) and 3-(4,5-dimethylthiazol-2-yl)-2,5 diphenyl tetrazolium bromide (MTT) assays (*p* = 0.094 and 0.453 for Piezo1 down/up-regulation, respectively), however, demonstrated that the viability of mASMCs was not affected by the down/up-regulation of Piezo1 (Figure S2B).

**Figure 3:**
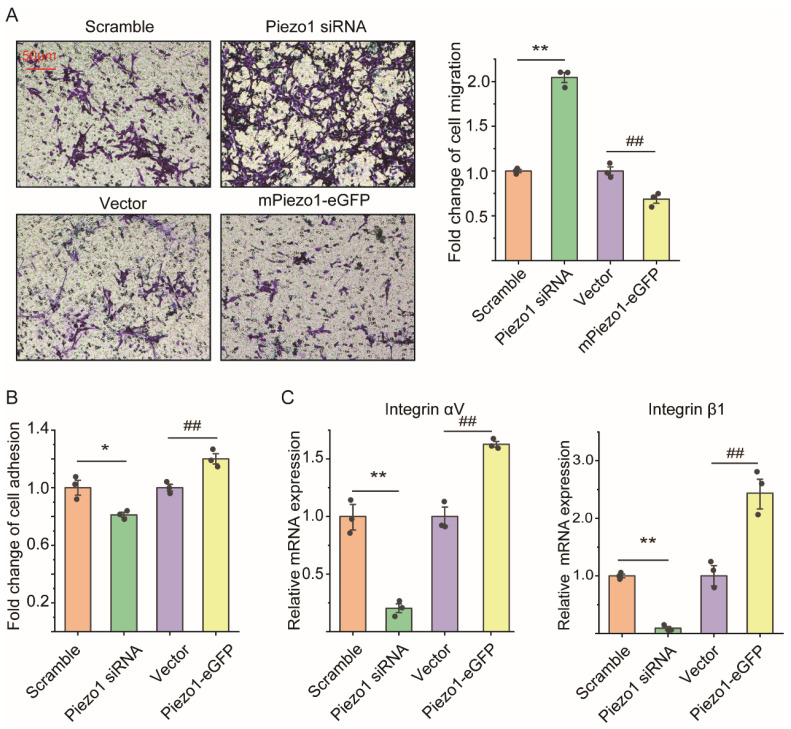
Effects of Piezo1 mRNA expression regulated by Piezo1 siRNA and Piezo1-eGFP transfection on cell migration, cell adhesion, and integrin mRNA expression of in vitro mASMCs. (**A**) Representative images (left) and quantification (right) of cell migration in transwell assay of mASMCs with Piezo1 expression either down-regulated, up-regulated, or unaltered by transfection of Piezo1 siRNA, Piezo1-eGFP, or Scramble and Vector. (**B**,**C**) Quantification of cell adhesion by cell adhesion assay, and mRNA expression of integrin (αV, β1) by qPCR of mASMCs transfected with either Piezo1 siRNA, Piezo1-eGFP, or Scramble and Vector. In all cases, n = 3. Markers denote significant differences from control conditions on the Scramble group (* *p* < 0.05, ** *p* < 0.01) or Vector group (^##^ *p* < 0.01).

Figure 3A shows representative images (left panel) and quantified results (right panel) of cell migration by transwell assay of mASMCs transfected with Piezo1 siRNA, mPiezo1-eGFP, Scramble, and Vector, respectively. Cell migration of mASMCs transfected with Piezo1 siRNA/mPiezo1-eGFP was obviously increased/decreased, as seen in the images, which was quantified as a ~1-fold increase (*p* = 0.001, Piezo1 siRNA vs. Scramble), and a ~40% decrease (*p* = 0.008, mPiezo1-eGFP vs. Vector), respectively. Figure 3B shows that cell adhesion of mASMCs transfected with Piezo1 siRNA/mPiezo1-GFP was decreased/increased by ~20% compared with those transfected with Scramble/Vector (*p* = 0.026 and *p* = 0.009 for Piezo1 siRNA and Piezo1-GFP groups, respectively). Figure 3C shows that mRNA expression of integrin αV (*p* = 0.007 and 0.002 for Piezo1 siRNA and Piezo1-GFP groups, respectively) and integrin β1 (*p* = 0.001 for both Piezo1 siRNA and Piezo1-GFP groups) in mASMCs transfected with Piezo1 siRNA/mPiezo1-GFP was decreased/increased to a large extent compared with those transfected with Scramble/Vector.

### 2.4. ROCK Signaling Inhibition Abolished the Cell Migration Due to Loss of Piezo1 mRNA of ASMCs

**Figure 4:**
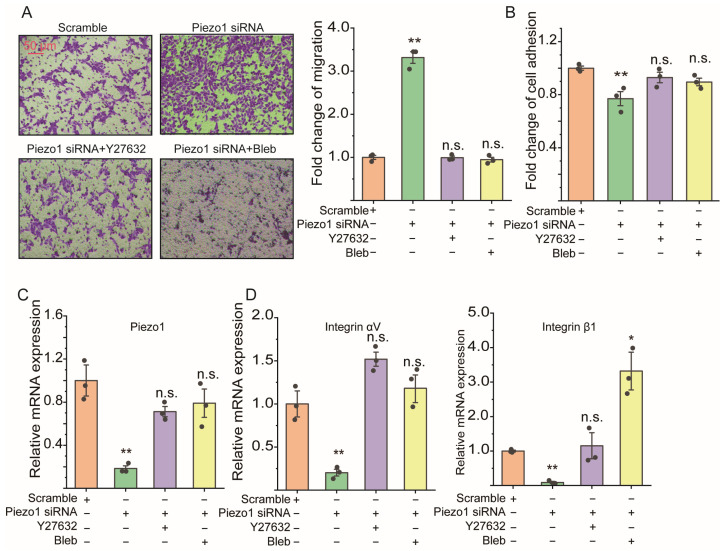
Effect of ROCK signaling inhibition on cell migration, cell adhesion, and integrin (αV, β1) mRNA expression of in vitro mASMCs. (**A**) Representative images (left) and quantification (right) of cell migration in transwell assay of mASMCs treated with either Piezo1 siRNA, Y27632, Bleb (myosin II inhibitor) to inhibit ROCK signaling or Scramble as control. (**B**–**D**) Quantification of cell adhesion by cell adhesion assay, mRNA expression of Piezo1 and integrin (αV, β1) by qPCR of mASMCs treated with either Piezo1 siRNA, Y27632, Bleb (myosin II inhibitor) to inhibit ROCK signaling or Scramble as control. In all cases, n = 3. Markers denote not significant (n.s.) or significant difference from static group (* *p* < 0.05, ** *p* < 0.01).

Figure S3 shows the effect of ROCK signaling inhibitor Y27632 (0.5, 1, and 5 μM, dissolved with DMSO) and myosin II inhibitor blebbistatin (Bleb, 5, 10, and 20 μM, dissolved with DMSO) on the cellular activity of mASMCs, evaluated with MTT and CCK-8. MTT results (Figure S3A) show that 0.5, 1, and 5 μM Y27632 and 5 and 10 μM Bleb treatment for 24 h did not affect cellular activity, but a high dose of Bleb (20 μM) significantly reduced cellular activity (*p* = 0.007). These results were confirmed with a CCK-8 assay (*p* = 0.002 for 20 μM Bleb). When mASMCs transfected with Piezo1 siRNA for 36 h were treated with ROCK signaling inhibitor Y27632 (1 μM) and myosin II inhibitor blebbistatin (Bleb, 10 μM) for an additional 12 h, the enhanced cell migration due to the loss of Piezo1 of mASMCs was completely abolished, as seen clearly in the representative images (Figure 4A, left panel) and quantified results (Figure 4A, right panel) of the transwell assay (*p* = 0.001, 0.912, and 0.507 for Piezo1 siRNA, Piezo1 siRNA+Y27632, Piezo1 siRNA+Bleb vs. Scramble, respectively). Figure 4B–D show that the reduced cell adhesion (*p* = 0.014, 0.175, and 0.313 for Piezo1 siRNA, Piezo1 siRNA+Y27632, Piezo1 siRNA+Bleb vs. Scramble, respectively), mRNA expression of Piezo1 (*p* = 0.037, 0.244, and 0.317 for Piezo1 siRNA, Piezo1 siRNA+Y27632, Piezo1 siRNA+Bleb vs. Scramble, respectively), and mRNA expressions of integrin αV (*p* = 0.007, 0.060, and 0.484 for Piezo1 siRNA, Piezo1 siRNA+Y27632, and Piezo1 siRNA+Bleb vs. Scramble, respectively) and integrin β1 (*p* = 0.001, 0.706, and 0.013 for Piezo1 siRNA, Piezo1 siRNA+Y27632, Piezo1 siRNA+Bleb vs. Scramble, respectively) of mASMCs due to a loss of Piezo1 mRNA was also reversed to various extents by ROCK signaling inhibitor Y27632 and myosin II inhibitor Bleb. Figure S4 shows that Y27632 (0.5, 1, 5 μM) and Bleb (1, 5, 10 μM) functioned in a dose-dependent manner with regard to the loss of Piezo1 mRNA-enhanced cell migration. However, high doses of them (5 μM Y27632 and 10 μM Bleb) did not further enhance the inhibitory effect on the loss of Piezo1-decreased cell adhesion.

### 2.5. Piezo1 Knockin and ROCK Signaling Inhibition Both Reversed the Cell Migration Enhancement of ASMCs Due to High Stretch

**Figure 5:**
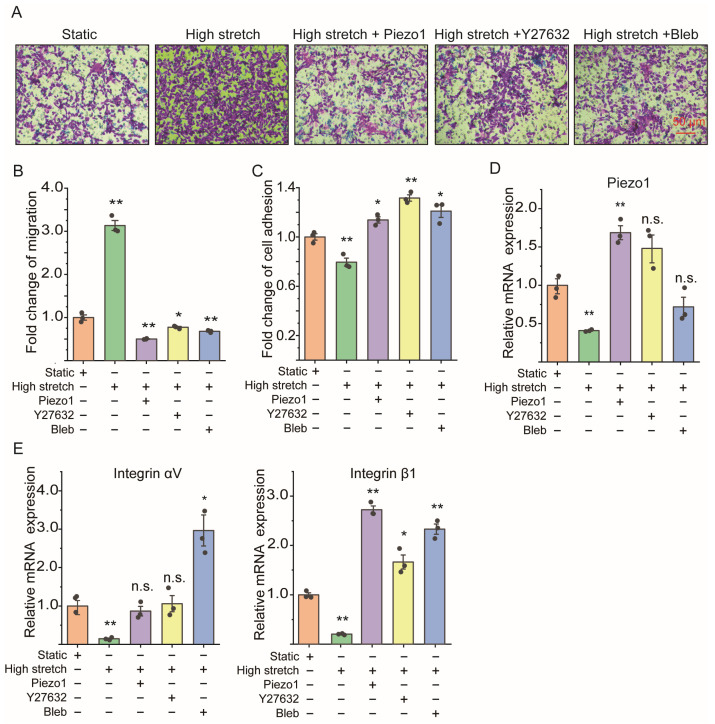
Effect of high stretch with/without Piezo1 knockin and ROCK signaling inhibition on cell migration, cell adhesion, mRNA expression of Piezo1, and integrin (αV, β1) of in vitro mASMCs. (**A**) Representative images of cell migration in transwell assay of mASMCs cultured in either static or high-stretch conditions with/without pretreatment of Piezo1 knockin, ROCK inhibitor Y27632, or myosin II inhibitor Bleb (Static, High stretch, High stretch+Piezo1, High stretch+Y27632, High stretch+Bleb, respectively). (**B**–**E**) Quantification of cell migration by transwell assay, cell adhesion by cell adhesion assay, mRNA expression of Piezo1 and integrin (αV, β1) by qPCR of ASMCs cultured in either static or high-stretch condition with/without pretreatment of Piezo1 knockin, ROCK inhibitor Y27632, or myosin II inhibitor Bleb (Static, High stretch, Piezo1, Y27632, Bleb, respectively). In all cases, n = 3. Markers denote not significant (n.s.) or significant difference from the static group (* *p* < 0.05, ** *p* < 0.01).

Figure 5 shows that Piezo1 knockin and ROCK signaling inhibition could both reverse the high stretch-induced cell migration enhancement, cell adhesion reduction, Piezo1, and integrin (αV, β1) mRNA expression down-regulation. As shown by the representative images of the transwell assay in Figure 5A, the high stretch-induced greater cell migration of mASMCs (see high stretch vs. static, *p* = 0.001) was apparently reduced when the highly stretched cells were also pretreated with Piezo1 knockin (*p* = 0.001), Y27632 (1 μM, *p* = 0.0256), or Bleb (10 μM, *p* = 0.007) for 1 h, respectively (see high stretch+Piezo1, high stretch+Y27632, high stretch+Bleb vs. high stretch). Figure 5B–D show quantitative results indicating that the high stretch-induced cell adhesion reduction (*p* = 0.008, 0.060, 0.0014, 0.018 for high stretch, high stretch+Piezo1 knockin, high stretch+Y27632, and high stretch+Bleb vs. static, respectively) and Piezo1 mRNA (*p* = 0.008, 0.012, 0.267, 0.196 for high stretch, high stretch+Piezo1 knockin, high stretch+Y27632, and high stretch+Bleb vs. static, respectively), integrin αV mRNA (*p* = 0.009, 0.634, 0.853, 0.031 for high stretch, high stretch+Piezo1 knockin, high stretch+Y27632, and high stretch+Bleb vs. static, respectively), and integrin β1 mRNA (*p* = 0.005, 0.001, 0.011, 0.001 for high stretch, high stretch+Piezo1 knockin, high stretch+Y27632, and high stretch+Bleb vs. static, respectively) down-regulation of mASMCs were all reversed to various extents by either Piezo1 knockin or the inhibition of ROCK signaling with ROCK signaling inhibitor Y27632 and myosin II inhibitor Bleb. Figure S5 shows that different doses of Y27632 (0.5, 1, 5 μM) have similar effects on high stretch-enhanced cell migration and -decreased cell adhesion, and Bleb (1, 5, 10 μM) functioned without a dose-dependent effect on the loss of Piezo1-enhanced cell migration and -decreased cell adhesion.

### 2.6. ROCK Signaling Inhibition Abolished the Cell Migration Enhancement and Piezo1 Reduction of ASMCs from Mice after MV

**Figure 6:**
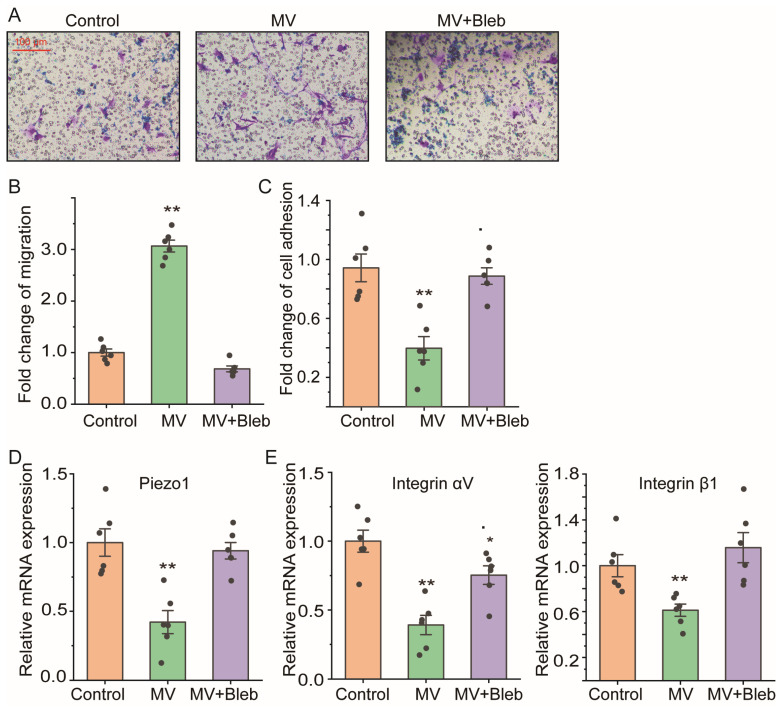
Effect of ROCK signaling inhibition on cell migration, cell adhesion, and mRNA expression of Piezo1 and integrin (αV, β1) of pASMCs isolated from mice after high-tidal-volume MV. (**A**) Representative images of cell migration in transwell assay of primary ASMCs (pASMCs) isolated from mice after either spontaneous breathing or mechanical ventilation at high tidal volume (VT 18 mL/kg, 3 h) and cultured in the absence or presence of myosin II inhibitor Bleb (Control, MV, MV+Bleb, respectively). (**B**–**E**) Quantification of cell migration by transwell assay, cell adhesion by cell adhesion assay, mRNA expression of Piezo1 and integrin (αV, β1) by qPCR of pASMCs in control, MV, and MV+Bleb groups. In all cases, n = 6. Markers denote not significant (n.s.) or significant difference from the control group (* *p* < 0.05, ** *p* < 0.01).

We further tested whether ROCK signaling actually mediated the in vivo effect of MV on ASMCs by measuring cell migration, cell adhesion, Piezo1, and integrin (αV, β1) mRNA expression of pASMCs isolated from mice after MV, with/without exposing mice to myosin II inhibitor Bleb (10 μM, 3 h). The representative images and quantitative results of cell migration in a transwell assay (Figure 6A,B) show that the increased cell migration of pASMCs isolated from mice after MV treated with Bleb was completely abolished (*p* = 0.001, MV vs. control; *p* = 0.001, MV+Bleb vs. control). In addition, ROCK signaling inhibition also reversed the reduction in cell adhesion (*p* = 0.001, MV vs. control; *p* = 0.139, MV+Bleb vs. control), Piezo1 (*p* = 0.001, MV vs. control; *p* = 0.621, MV+Bleb vs. control), and integrin αV (*p* = 0.001, MV vs. control; *p* = 0.041, MV+Bleb vs. control) and integrin β1 (*p* = 0.005, MV vs. control; *p* = 0.357, MV+Bleb vs. control) mRNA expression to various extents (Figure 6C–E).

## 3. Discussion

This study demonstrates that in mouse models of MV at high tidal volume, Piezo1 and integrin mRNA expression were greatly reduced in lung tissue and pASMCs compared to those without MV. Perhaps more importantly, the pASMCs from mice treated with MV showed an enhanced ability to migrate but reduced cell adhesion when cultured and tested for cell migration and adhesion in vitro. All the cellular changes of ASMCs in response to MV in vivo or MV-associated high stretch in vitro, in particular the enhanced cell migration together with reduced Piezo1 and integrin mRNA expression and cell adhesion, could be either induced by down-regulation of Piezo1 mRNA in the cells with siRNA, or abolished by Piezo1 knockin and ROCK signaling inhibition. These findings suggest that high stretch associated with MV is a potent mechanical modulator of Piezo1 mRNA expression in ASMCs. Through this mechanical modulation of Piezo1 mRNA expression and ROCK signaling mediation, the mechanical stimulation on airways due to MV promotes cell migration by suppressing Piezo1 and integrin mRNA expression as well as cell adhesion of ASMCs. This may be a novel mechanism of detrimental airway remodeling occurring in ARDS patients under MV, and ultimately, a contributor to VILI, and, thus, may provide a potential therapeutic target for preventing/treating the clinically challenging VILI.

Previous research has demonstrated that VILI is attributable to high stretch during MV [15]. Many patients with MV survive the acute phase but subsequently die with evidence of significant progressive fibrosis [36]. Such fibrosis can be attributed to a resource of diverse cell types including fibroblasts, myofibroblasts, as well as smooth muscle cells [37,38]. In this study, we found that ASMCs from mice treated with high-tidal-volume MV and in culture treated with high stretch both showed an enhanced ability to migrate, suggesting that ASMCs provide an important cell source that can be mobilized to contribute to the airway fibrotic process during MV.

Although stretch has been shown to enhance the cell migration velocity of several types of cells including osteoblasts and tenocytes, the underlying mechanism remains not fully understood [39]. During cell migration, cells can change migration velocity during the transition between different migration modes depending on various factors such as integrin, confinement, contractility, and adhesion [23]. Previously, we have shown that Piezo1 mediates cell migration of breast cancer cells in response to compression [40]. Here, we show that Piezo1 could also dramatically mediate cell migration of ASMCs in response to MV and high stretch, in a negatively dependent manner. Consistently, the cell migration of ASMCs induced by MV/high stretch was negatively correlated with integrin mRNA expression and cell adhesion, suggesting that Piezo1 is mechanically modulated to mediate cell migration of ASMCs.

Furthermore, there is ample evidence of cross-talk between Piezo1 and integrin to modulate the cell migration mode, which may play an important role in determining the migration behavior of ASMCs in response to stretch [41,42]. For example, in tumor epithelial cells and immune cells, it is known that the loss of Piezo1 induces a fast integrin-independent migration together with the modulation of integrin expression and cell adhesion [22,24,25,26,29]. In A549 cells (a human alveolar epithelial cell line), Piezo1 is increased during high-tidal-volume MV and cyclic stretch [34]. On the contrary, we found in ASMCs that Piezo1 mRNA was decreased with increased cell migration during high-tidal-volume MV and cycling. Taken together, we believe that the loss of Piezo1 in ASMCs due to MV or high stretch promoted a fast integrin-independent migration.

Fast integrin-independent migration involves both local protrusion and global cortical flow that result from myosin II-dependent mechanical instability of the cell cortex [23]. Since ROCK signaling is well known to activate myosin II to influence localized cell contractility and to be activated in response to stretch, it is highly likely to promote an integrin-independent migration during MV [22,43,44]. In this work, we found that ROCK and myosin II inhibition did abolish the cell migration of ASMCs induced by high stretch, which further supports the view that high stretch induces fast integrin-independent migration by activating myosin II in ASMCs.

The present study has some limitations, though. First, the expression changes of Piezo1 and integrin (αV, β1) in lung tissue and ASMCs in vivo and in vitro were mainly detected at the mRNA level with qPCR, which needs to be confirmed at the protein level by Western blotting. Second, we cannot exclude the possible impact of low integrin affinity due to the low expression of Piezo1 on cell migration of ASMCs. Third, we cannot prove that target genes of the Piezo1 pathway in vivo contribute to the pathogenesis of airway fibrosis in VILI because we did not use Piezo1 overexpressed animal models in this study. Finally, airway epithelial cells are not considered in this study, even though they are equally as, if not more, important as ASMCs in lung injury and repair during MV. Therefore, there are still many questions to be resolved before we can fully understand the role of Piezo1 in VILI.

## 4. Materials and Methods

### 4.1. Animals with Mechanical Ventilation

Female C57BL/6 mice (4–8 weeks, weight 18–22 g) were purchased from Cavens Lab Animal Co., Ltd. (Changzhou, China) and housed in specific pathogen-free conditions at ~25 °C of room temperature and 50–60% relative humidity, provided with a 12 h light/dark cycle and free access to food and water. All the animal experiment protocols were approved by the Biomedical Research Ethics Committee of Changzhou University following the Institutional Guidelines for Animal Care and Use (license No. SCXK-(JS)-2021-0013, Changzhou, China).

Before the experiment, the mice were randomly divided into three groups (n = 6 per group) for testing, namely, a group with spontaneous breathing (control), a group with high-tidal-volume mechanical ventilation (MV), and a group with MV followed by isolation and treatment of ASMCs with 1.2 mg/kg myosin II inhibitor blebbistatin (Bleb+MV, 10 μM, #HY-13441, MCE, Shanghai, China). The procedure for mechanical ventilation of mice was modified from that previously described [45]. Briefly, mice were anesthetized with intraperitoneal injection of pentobarbitone (50 mg/kg). Then the trachea of each mouse was intubated with a 14G Teflon catheter and connected to the adapter of the instrument. Thereafter, animals were connected to a time-cycled, volume-limited FlexiVent system (SciReq, Montreal, PQ, Canada) and ventilated for 3 h with room air at high tidal volume (V_T_, 18 mL/kg, and 2 cm H_2_O positive end-expiratory pressure, PEEP). The respiratory rate was set at 30 breaths/minute. Preliminary studies showed that these respiratory settings resulted in normal PaCO_2_ values after 3 h of MV. At the end of MV, animals were killed by exsanguination after supplemental pentobarbital (10 mg/kg).

### 4.2. Histological Evaluation of Piezo1 Expression in Mouse Lung

Histological evaluation of Piezo1 expression in mouse lungs was carried out following a previously described procedure with modification [46]. Briefly, mice, after 3 h MV, were operated on with a midline thoracotomy to excise the lungs. Then, the lungs were fixed by intratracheal instillation of 0.5 mL of 10% neutral buffered formalin, followed by floating in 10% formalin for a week. Lung specimens were embedded in paraffin and serially sliced from apex to base to obtain 5 μm thick lung tissue sections. The sections were then de-paraffinized and hydrated in xylene and graded ethanol before being stained with hematoxylin-eosin (HE) and immunostaining antibody against Piezo1. The latter is described briefly in the following. After antigen retrieval in sodium citrate and 3% H_2_O_2_ blocking of endogenous peroxidase, lung tissue sections were sealed with 5% normal goat serum for 30 min at room temperature (RT). Subsequently, they were incubated with primary antibody against Piezo1 (1:100) overnight at 4 °C, then with horseradish peroxidase (HRP)-conjugated goat anti-rabbit antibody for 1 h and DAB for color development. Afterward, they were viewed and photographed at ×20 objective using a light microscope with a camera (STL-FL4CKX53LED, SITUOLI, Nanjing, China).

### 4.3. Isolation of Primary Mouse ASMCs from Mice after MV

Primary mouse ASMCs (pASMCs) were isolated from C57BL/6 mice after either spontaneous breathing or MV following a previously described procedure with modification [47]. Briefly, C57BL/6 mice were anesthetized with intraperitoneally injected pentobarbitone (50 mg/kg), and then cells were quickly removed from the tracheas and mainstem bronchi to be placed in a pre-chilled dissociation solution consisting of (in mM): 135 NaCl, 6 KCl, 5 MgCl_2_, 0.1 CaCl_2_, 0.2 EDTA, 10 HEPES, and 10 Glucose (pH 7.3). Subsequently, small dices of airway tissue dissected from the tracheas and mainstem bronchi after clearing the connective tissue were incubated in the dissociation medium containing 0.2 mg/mL papain (#P4762, Sigma, St. Louis, MO, USA), 8.53 mg/mL type I collagenase (#C0130, Sigma), 0.14 mg/mL DL-Dithiothreitol (DTT, #D806827, Macklin, Shanghai, China), and 2 mg/mL BSA, at 37 °C for 40 min. Afterward, the tissue was agitated with a fire-polished wide-bore glass pipette to release the cells that were finally verified as pASMCs by immunostaining using the antibody against alpha-smooth muscle actin (α-SMA, marker of ASMCs) (#BM0002, Boster Biological Technology Co., Ltd., Wuhan, China), and the typical “hill and valley” appearance under phase contrast microscopy of the cells in culture. These pASMCs were then used for qPCR, cell adhesion, and migration assay.

### 4.4. Assessment of Piezo1 Expression and Distribution in pASMCs

Piezo1 expression and distribution in pASMCs were assessed by immunofluorescent staining. In brief, cells were fixed in 3.7% paraformaldehyde for 15 min at RT, then permeabilized with 0. 5% Tween-X-100 for 15 min at RT. Nonspecific binding of antibody was blocked by incubating the samples in phosphate-buffered saline (PBS) containing 1% BSA for 1 h at RT. Piezo1 was stained by incubating the cells first with anti-Piezo1 antibodies (#PA5-72974, Thermo Fisher, Waltham, MA, USA) overnight at 4 °C, then with goat anti-mouse IgG (H+L) cross-adsorbed secondary antibody Alexa FluorTM 546 (#A-11035, Thermo Fisher) for 1 h at RT in the dark. The nucleus was stained with 4′,6-diamidino-2-phenylindole (DAPI, #C1005, Beyotime Biotechnology, Shanghai, China) for 10 min. Between all staining steps, cells were washed 3 times with PBS. Stained cells were visualized with laser scanning confocal microscopy (LSM710, Carl Zeiss, Jena, Germany) using oil objective (×63). Acquisition parameters were kept constant during the experiment. Fluorescent images were processed with ImageJ 1.53t software.

### 4.5. Culture of Cell Line Mouse ASMCs with High Stretch

Cell line mouse ASMCs (mASMCs, #BNCC359490, BeNa Culture Collection, Beijing, China) were purchased from BeNa Culture Collection (Beijing, China) and cultured in Dulbecco’s modified Eagle’s medium (DMEM, #11885092, Thermo Fisher) supplemented with 10% fetal bovine serum (FBS, #16000-044, Thermo Fisher), 2 mM L-glutamine, 100 units/mL penicillin, and 100 μg/mL streptomycin in an incubator containing 5% CO_2_ humidified at 37 °C according to the method described previously [20,21,35]. mASMCs were identified with immunostaining antibodies against α-SMA (#BM0002). mASMCs in passages 3–8 showing typical “hill and valley” morphology and abundant α-SMA expression were used for experiments.

For the high-stretch experiment, 2 × 10^4^ cells/cm^2^ mASMCs in exponential proliferation were plated on type I collagen-coated Bioflex 6-well plates and were serum-deprived for 24 h. Subsequently, we exposed the cells grown on Bioflex plates to a 13% cyclic strain at 0.5 Hz for 72 h (a sinusoidal wave, 1 s of deformation alternating with 1 s of relaxation) using the Flexcell FX-5000 system (Flexcell International, Hillsborough, NC, USA) to simulate MV-associated high stretch on ASMCs as previously described [4,20,21,48]. The mASMCs grown on Bioflex plates in static condition for 72 h were used as controls. After high-stretch treatment, mASMCs were analyzed for cell migration by transwell assay, cell adhesion by cell adhesion assay, and mRNA expression of Piezo1 and integrin by qPCR.

### 4.6. Assessment of Cell Migration of ASMCs

Cell migration of ASMCs (pASMCs, mASMCs) after treatment with high stretch or Piezo1 modulation with or without ROCK inhibitor Y27632 (#HY-10071, MCE, Shanghai, China) or myosin II inhibitor Bleb was measured by using a 6-well transwell chamber that was separated into upper and lower compartments by a filter membrane with 8 μm pores (#07-200-169, Corning, New York, NY, USA). Briefly, ASMCs were plated in the upper compartment (1 × 10^5^ cells/well), and replaced with serum-free medium after 12 h, while the lower compartment was filled with 2 mL complete medium. After 24 h, the cells were fixed with 4% paraformaldehyde (#30525-89-4, Electron Microscopy Sciences, Hatfield, PA, USA). The non-invasive cells in the upper compartment were removed with cotton swabs, and the invaded cells in the lower compartment were stained with 0.1% crystal violet (#C6158, Sigma) for 10 min at RT. Afterward, the stained cells in the lower compartment were examined and counted by light microscopy at 10× objective (Olympus BX60, Olympus Corp., Tokyo, Japan) and ImageJ software. For each compartment, 10 random fields were analyzed for their cell number and each experiment was repeated three times.

### 4.7. Assessment of Cell Adhesion of ASMCs

Cell adhesion of ASMCs (pASMCs, mASMCs) was measured by using a cell adhesion assay as previously described [49]. Briefly, 2 × 10^5^ cells were seeded into plates coated with poly-l-lysine (10 μg/mL for 60 min, 37 °C). After washing with PBS to remove non-adherent cells, the adherent cells were fixed (3% paraformaldehyde, 5 min) and stained with methylene blue (0.4%, 5 min). Cells were washed three times with PBS, and HCl (0.1 M) was used to eluate intracellular methylene blue. Then, the optical density (630 nm) of each sample was measured with a plate reader (Infinite F50, TECAN, Männedorf, Switzerland). Cell adhesion per condition was expressed as a percentage of total cellular adhesion to control groups.

### 4.8. Assessment of mRNA Expression in ASMCs

The mRNA expression of Piezo1 and integrin αVβ1 in ASMCs (pASMCs, mASMCs) were evaluated with qPCR. The associated primers were purchased from General Biosystems (Chuzhou, China, Table S1). Briefly, total RNA from ASMCs was purified using TRI Reagent RNA Isolation Reagent (#T9424, Sigma), and the RNA was quantified with a Nanodrop 2000 spectrophotometer (Thermo Scientific, Willmington, DE, USA). Then, 500 ng RNA was used to generate 1st-strand cDNA with the RevertAid First Strand cDNA Synthesis Kit (#K1622, Thermo Scientific, MA, USA). PCR was performed with PowerUp SYBR Green Master Mix (#A25742, Applied Biosystems, Foster City, CA, USA) using the StepOne real-time PCR system (Applied Biosystems) at 50 °C for 2 min, 95 °C for 2 min, followed by 40 cycles of 95 °C for 15 s, 55 °C for 15 s, and 72 °C for 60 s. The reaction system contained 1 µL of cDNA in a 10 µL reaction and was prepared in triplicate. Calibration was done using the 2^−∆∆CT^ method, where ∆C_T_ = C_T_ (target gene) − C_T_ (GAPDH) and ∆∆C_T_ = ∆C_T_ (experiment) − ∆C_T_ (control). Fold changes in different genes were calculated as the ratio of experiments to the controls from the resulting 2^−∆∆CT^ values.

### 4.9. Transfection of Piezo1 siRNA/Piezo1-eGFP into mASMCs

The Piezo1 mRNA expression in mASMCs was modulated by transfection of Piezo1 siRNA or Piezo1-eGFP into the cells. Piezo1 siRNA (shown in Table S1) and Piezo1-eGFP (#80925) were obtained from General Biosystems (Chuzhou, China) and Addgene (Watertown, MA, USA), respectively. The cells were transfected following the manufacturer’s instructions. Briefly, 2 × 10^4^ cells/cm^2^ mASMCs were plated on type I collagen-coated plates to 50–80% confluence and then serum-deprived for 24 h. Subsequently, the cells were transfected with either Piezo1 siRNA, Piezo1-eGFP, or the Scramble control by Lipofectamine 3000 reagent (#L3000015, Thermo Fisher Scientific) and incubated for 12 h in FBS-free Opti-MEM medium. Then, the cells were incubated in FBS-free DMEM medium for another 72 h before other experiments. The transfection efficiency was verified by qPCR.

### 4.10. Assessment of Viability of ASMCs

Cell activity of mASMCs transfected with Y27632, Bleb, Piezo1 siRNA, mPiezo1-eGFP, Scramble, or Vector were evaluated with a CCK-8 assay kit (#C0040, Beyotime Biotechnology) and 3-(4,5-dimethylthiazol-2-yl)-2,5 diphenyl tetrazolium bromide (MTT, #ST316, Beyotime Biotechnology) assays as described previously [50]. Briefly, cells were plated in 96-well plates at a density of 1 × 10^4^ cells/well. After overnight cultivation, the cells were transfected for 48 h, and then 10 μL of CCK-8 or MTT solution was added to the cells for 4 h at 37 °C. For the CCK-8 assay, absorbance was acquired by using an automatic microplate reader (Infinite F50, Tecan, Männedorf, Switzerland) at 450 nm. For the MTT assay, the sample’s supernatant was discarded and replaced with 150 μL DMSO, and the sample was shaken with a shaking table at 150 rpm/min for 3 min. Absorbance was acquired by using an automatic microplate reader (Infinite F50) at 492 nm.

### 4.11. Statistical Analysis

Statistical analysis was performed by using GraphPad Prism 9.0 (Graph Pad Software, La Jolla, CA, USA). All data followed normal distribution as verified by distribution test using Origin 2018 (OriginLab Corporation, Northampton, MA, USA), and, thus, reported as means ± S.E.M. Unless stated otherwise, each sample was tested in triplicate, and each experiment for a given study condition was repeated the same number of times as the group size (n). One-way analysis of variance (ANOVA) followed by a post-ad hoc Student’s *t*-test was carried out for multiple comparisons using Origin 2018. *p* < 0.05 was considered statistically significant.

## 5. Conclusions

We demonstrate that high stretch in both in vivo and in vitro models led to decreased mRNA expression of Piezo1 and integrin (αV, β1) in ASMCs, together with enhanced cell migration but reduced cell adhesion. Artificial down-regulation of Piezo1 mRNA in ASMCs by siRNA could also lead to decreased expression of integrin (αV, β1), enhanced cell migration, and impaired cell adhesion. Taken together, high-tidal-volume MV and associated high stretch could decrease Piezo1 mRNA expression to promote fast integrin-independent migration of ASMCs. This observation of Piezo1-mediated cell migration during high stretch provides a novel mechanism and corresponding new therapeutic target for the prevention and management of detrimental airway remodeling in pulmonary diseases such as VILI.

## Data Availability

The raw data supporting the conclusions of this article will be made available by the authors, without undue reservation.

## Supplementary Materials

The following supporting information can be downloaded at: https://www.mdpi.com/article/10.3390/ijms25031748/s1.

## References

[1] Plataki M., Hubmayr R.D. (2010). The physical basis of ventilator-induced lung injury. Expert Rev. Respir. Med..

[2] Sinclair S.E., Molthen R.C., Haworth S.T., Dawson C.A., Waters C.M. (2007). Airway strain during mechanical ventilation in an intact animal model. Am. J. Respir. Crit. Care Med..

[3] Ibrahim I.B.M., Aghasafari P., Pidaparti R.M. (2016). Transient mechanical response of lung airway tissue during mechanical ventilation. Bioengineering.

[4] Retamal J., Hurtado D., Villarroel N., Bruhn A., Bugedo G., Amato M.B.P., Costa E.L.V., Hedenstierna G., Larsson A., Borges J.B. (2018). Does regional lung strain correlate with regional inflammation in acute respiratory distress syndrome during nonprotective ventilation? An experimental porcine study. Crit. Care Med..

[5] Villar J., Cabrera N.E., Valladares F., Casula M., Flores C., Blanch L., Quílez M.E., Santana-Rodríguez N., Kacmarek R., Slutsky A.S. (2011). Activation of the wnt/β-catenin signaling pathway by mechanical ventilation is associated with ventilator-induced pulmonary fibrosis in healthy lungs. PLoS ONE.

[6] D’Angelo E., Koutsoukou A., Valle P.D., Gentile G., Pecchiari M. (2008). Cytokine release, small airway injury, and parenchymal damage during mechanical ventilation in normal open-chest rats. J. Appl. Physiol..

[7] Xu H., Pan G., Wang J. (2023). Repairing mechanisms for distal airway injuries and related targeted therapeutics for chronic lung diseases. Cell Transplant..

[8] Garfield B., Handslip R., Patel B.V. (2021). Ventilator-associated lung injury. Encycl. Respir. Med..

[9] Cabrera-Benítez N.E., Parotto M., Post M., Han B., Spieth P.M., Cheng W.E., Valladares F., Villar J., Liu M., Sato M. (2012). Mechanical stress induces lung fibrosis by epithelial-mesenchymal transition. Crit. Care Med..

[10] Cullen A.B., Cooke P.H., Driska S.P., Wolfson M.R., Shaffer T.H. (2006). The impact of mechanical ventilation on immature airway smooth muscle: Functional, structural, histological, and molecular correlates. Biol. Neonate.

[11] Hislop A.A., Haworth S.G. (1990). Pulmonary vascular damage and the development of cor pulmonale following hyaline membrane disease. Pediatr. Pulmonol..

[12] Albertine K.H., Jones G.P., Starcher B.C., Bohnsack J.F., Davis P.L., Cho S.C., Carlton D.P., Bland R.D. (1999). Chronic lung injury in preterm lambs. Disordered respiratory tract development. Am. J. Respir. Crit. Care Med..

[13] Sward-Comunelli S.L., Mabry S.M., Truog W.E., Thibeault D.W. (1997). Airway muscle in preterm infants: Changes during development. J. Pediatr..

[14] Hasaneen N.A., Zucker S., Cao J., Chiarelli C., Panettieri R.A., Foda H.D. (2005). Cyclic mechanical strain-induced proliferation and migration of human airway smooth muscle cells: Role of emmprin and mmps. FASEB J..

[15] López-Martínez C., Huidobro C., Albaiceta G.M., López-Alonso I. (2017). Mechanical stretch modulates cell migration in the lungs. Ann. Transl. Med..

[16] Coste B., Mathur J., Schmidt M., Earley T.J., Ranade S., Petrus M.J., Dubin A.E., Patapoutian A. (2010). Piezo1 and piezo2 are essential components of distinct mechanically activated cation channels. Science.

[17] Bavi N., Richardson J., Heu C., Martinac B., Poole K. (2019). Piezo1-mediated currents are modulated by substrate mechanics. ACS Nano.

[18] Ellefsen K.L., Holt J.R., Chang A.C., Nourse J.L., Arulmoli J., Mekhdjian A.H., Abuwarda H., Tombola F., Flanagan L.A., Dunn A.R. (2019). Myosin-ii mediated traction forces evoke localized piezo1-dependent Ca^2+^ flickers. Commun. Biol..

[19] Palecek S.P., Loftus J.C., Ginsberg M.H., Lauffenburger D.A., Horwitz A.F. (1997). Integrin-ligand binding properties govern cell migration speed through cell-substratum adhesiveness. Nature.

[20] Wen K., Ni K., Guo J., Bu B., Liu L., Pan Y., Li J., Luo M., Deng L. (2022). MircroRNA let-7a-5p in airway smooth muscle cells is most responsive to high stretch in association with cell mechanics modulation. Front. Physiol..

[21] Yang C., Guo J., Ni K., Wen K., Qin Y., Gu R., Wang C., Liu L., Pan Y., Li J. (2023). Mechanical ventilation-related high stretch mainly induces endoplasmic reticulum stress and thus mediates inflammation response in cultured human primary airway smooth muscle cells. Int. J. Mol. Sci..

[22] Carragher N.O., Walker S.M., Scott Carragher L.A., Harris F., Sawyer T.K., Brunton V.G., Ozanne B.W., Frame M.C. (2006). Calpain 2 and src dependence distinguishes mesenchymal and amoeboid modes of tumour cell invasion: A link to integrin function. Oncogene.

[23] Liu Y.J., Le Berre M., Lautenschlaeger F., Maiuri P., Callan-Jones A., Heuzé M., Takaki T., Voituriez R., Piel M. (2015). Confinement and low adhesion induce fast amoeboid migration of slow mesenchymal cells. Cell.

[24] Gudipaty S.A., Lindblom J., Loftus P.D., Redd M.J., Edes K., Davey C.F., Krishnegowda V., Rosenblatt J. (2017). Mechanical stretch triggers rapid epithelial cell division through piezo1. Nature.

[25] Solis A.G., Bielecki P., Steach H.R., Sharma L., Harman C.C.D., Yun S., de Zoete M.R., Warnock J.N., To S.D.F., York A.G. (2019). Mechanosensation of cyclical force by piezo1 is essential for innate immunity. Nature.

[26] McHugh B.J., Buttery R., Lad Y., Banks S., Haslett C., Sethi T. (2010). Integrin activation by fam38a uses a novel mechanism of r-ras targeting to the endoplasmic reticulum. J. Cell Sci..

[27] Yao M., Tijore A., Cheng D., Li J.V., Hariharan A., Martinac B., Tran Van Nhieu G., Cox C.D., Sheetz M. (2022). Force- and cell state-dependent recruitment of piezo1 drives focal adhesion dynamics and calcium entry. Sci. Adv..

[28] Cheng D., Wang J., Yao M., Cox C.D. (2023). Joining forces: Crosstalk between mechanosensitive piezo1 ion channels and integrin-mediated focal adhesions. Biochem. Soc. Trans..

[29] McHugh B.J., Murdoch A., Haslett C., Sethi T. (2012). Loss of the integrin-activating transmembrane protein fam38a (piezo1) promotes a switch to a reduced integrin-dependent mode of cell migration. PLoS ONE.

[30] Yang X.N., Lu Y.P., Liu J.J., Huang J.K., Liu Y.P., Xiao C.X., Jazag A., Ren J.L., Guleng B. (2014). Piezo1 is as a novel trefoil factor family 1 binding protein that promotes gastric cancer cell mobility in vitro. Dig. Dis. Sci..

[31] Ridley A.J. (2001). Rho gtpases and cell migration. J. Cell Sci..

[32] Xie N., Xiao C., Shu Q., Cheng B., Wang Z., Xue R., Wen Z., Wang J., Shi H., Fan D. (2023). Cell response to mechanical microenvironment cues via rho signaling: From mechanobiology to mechanomedicine. Acta Biomater..

[33] Tu P.C., Pan Y.L., Liang Z.Q., Yang G.L., Wu C.J., Zeng L., Wang L.N., Sun J., Liu M.M., Yuan Y.F. (2022). Mechanical stretch promotes macrophage polarization and inflammation via the rhoa-rock/nf-κb pathway. BioMed Res. Int..

[34] Zhang Y., Jiang L., Huang T., Lu D., Song Y., Wang L., Gao J. (2021). Mechanosensitive cation channel piezo1 contributes to ventilator-induced lung injury by activating rhoa/rock1 in rats. Respir. Res..

[35] Luo M., Ni K., Gu R., Qin Y., Guo J., Che B., Pan Y., Li J., Liu L., Deng L. (2023). Chemical activation of piezo1 alters biomechanical behaviors toward relaxation of cultured airway smooth muscle cells. Biol. Pharm. Bull..

[36] Cabrera-Benitez N.E., Laffey J.G., Parotto M., Spieth P.M., Villar J., Zhang H., Slutsky A.S. (2014). Mechanical ventilation-associated lung fibrosis in acute respiratory distress syndrome: A significant contributor to poor outcome. Anesthesiology.

[37] Todd N.W., Luzina I.G., Atamas S.P. (2012). Molecular and cellular mechanisms of pulmonary fibrosis. Fibrogenesis Tissue Repair.

[38] Zou F., Li Y., Zhang S., Zhang J. (2022). Dp1 (prostaglandin d(2) receptor 1) activation protects against vascular remodeling and vascular smooth muscle cell transition to myofibroblasts in angiotensin ii-induced hypertension in mice. Hypertension.

[39] Takemoto F., Uchida-Fukuhara Y., Kamioka H., Okamura H., Ikegame M. (2023). Mechanical stretching determines the orientation of osteoblast migration and cell division. Anat. Sci. Int..

[40] Luo M., Cai G., Ho K.K.Y., Wen K., Tong Z., Deng L., Liu A.P. (2022). Compression enhances invasive phenotype and matrix degradation of breast cancer cells via piezo1 activation. BMC Mol. Cell Biol..

[41] Li J., Hou B., Tumova S., Muraki K., Bruns A., Ludlow M.J., Sedo A., Hyman A.J., McKeown L., Young R.S. (2014). Piezo1 integration of vascular architecture with physiological force. Nature.

[42] Pathak M.M., Nourse J.L., Tran T., Hwe J., Arulmoli J., Le D.T., Bernardis E., Flanagan L.A., Tombola F. (2014). Stretch-activated ion channel piezo1 directs lineage choice in human neural stem cells. Proc. Natl. Acad. Sci. USA.

[43] Sahai E., Marshall C.J. (2003). Differing modes of tumour cell invasion have distinct requirements for rho/rock signalling and extracellular proteolysis. Nat. Cell. Biol..

[44] Wolf K., Mazo I., Leung H., Engelke K., von Andrian U.H., Deryugina E.I., Strongin A.Y., Brocker E.B., Friedl P. (2003). Compensation mechanism in tumor cell migration: Mesenchymal-amoeboid transition after blocking of pericellular proteolysis. J. Cell. Biol..

[45] Jiang L., Zhang Y., Lu D., Huang T., Yan K., Yang W., Gao J. (2021). Mechanosensitive piezo1 channel activation promotes ventilator-induced lung injury via disruption of endothelial junctions in ARDS rats. Biochem. Biophys. Res. Commun..

[46] Hübner R.H., Gitter W., El Mokhtari N.E., Mathiak M., Both M., Bolte H., Freitag-Wolf S., Bewig B. (2008). Standardized quantification of pulmonary fibrosis in histological samples. BioTechniques.

[47] Zhang C., Lifshitz L.M., Uy K.F., Ikebe M., Fogarty K.E., ZhuGe R. (2013). The cellular and molecular basis of bitter tastant-induced bronchodilation. PLoS Biol..

[48] Bagchi A., Vidal Melo M.F. (2018). Follow the voxel-a new method for the analysis of regional strain in lung injury. Crit. Care Med..

[49] Humphries M.J., Even-Ram S., Artym V. (2009). Cell adhesion assays. Extracellular Matrix Protocols.

[50] Jin Y., Liu L., Yu P., Lin F., Shi X., Guo J., Che B., Duan Y., Li J., Pan Y. (2021). Emergent differential organization of airway smooth muscle cells on concave and convex tubular surface. Front. Mol. Biosci..

