# Supplementary material for: High Stretch Associated with Mechanical Ventilation Promotes Piezo1-Mediated Migration of Airway Smooth Muscle Cells
Source: Int J Mol Sci. 2024 Feb 1;25(3):1748. doi: 10.3390/ijms25031748 (PMC10855813; doi:10.3390/ijms25031748)
Supplement: Supplementary file 1 [file ijms-25-01748-s001.zip › ijms-2790933-supplementary.pdf]

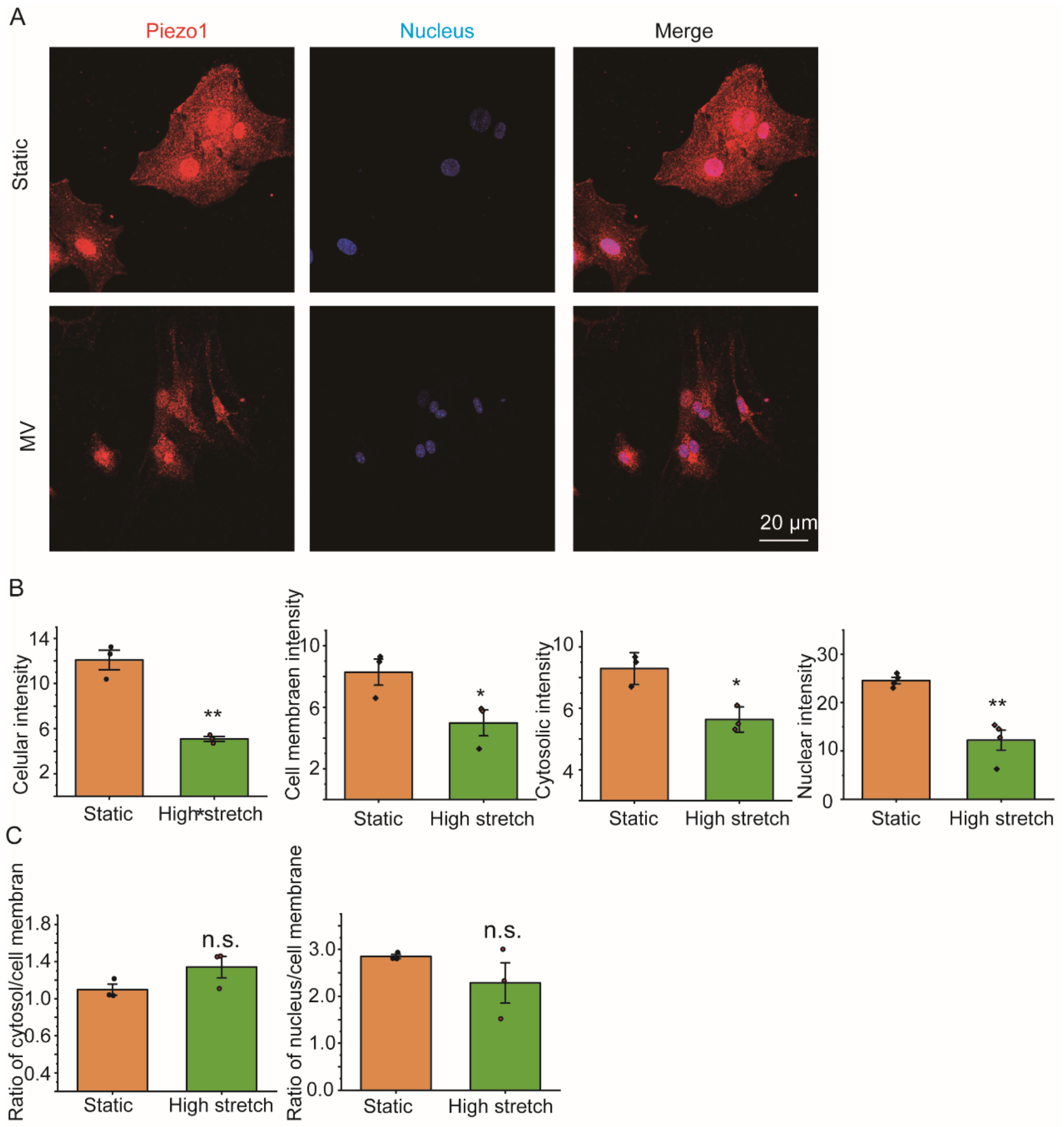

Figure S1: Piezo1 distribution in mouse primary airway smooth muscle cells (pASMCs) from spontaneous breathing (Control) or mechanical ventilation (MV). (**A**) Representative images of Piezo1 expression and distribution in primary airway smooth muscle cells (pASMCs) isolated from mice under either Control or MV at high vital volume (V_T, 18 ml/kg, 3 h), as evaluated with immunofluorescence assay. Bar = 20 μM. (**B**) The fluorescence intensity analysis of Piezo1 in pASMCs with ImageJ. (**C**) The ratio of cytosol intensity (left panel) or nucleus intensity (right panel) to cell membrane intensity. n = 3. **P* < 0.05; ** *P* < 0.01; n.s., not significant.

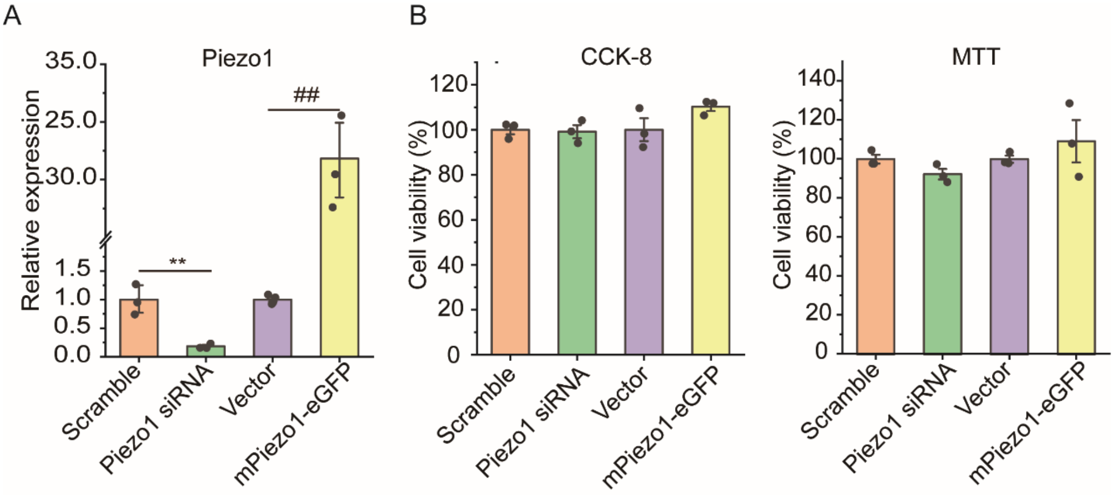

Figure S2: Piezo1 mRNA expression quantified by quantitative PCR (qPCR) (**A**) and cellular activity evaluated by CCK-8 or MTT assay (**B**) in cell line mouse ASMCs (mASMCs) transfected with either Piezo1 siRNA, mPiezo1-eGFP, Scramble, or Vector. n = 3. ** $P < 0.05$; ## $P < 0.01$.

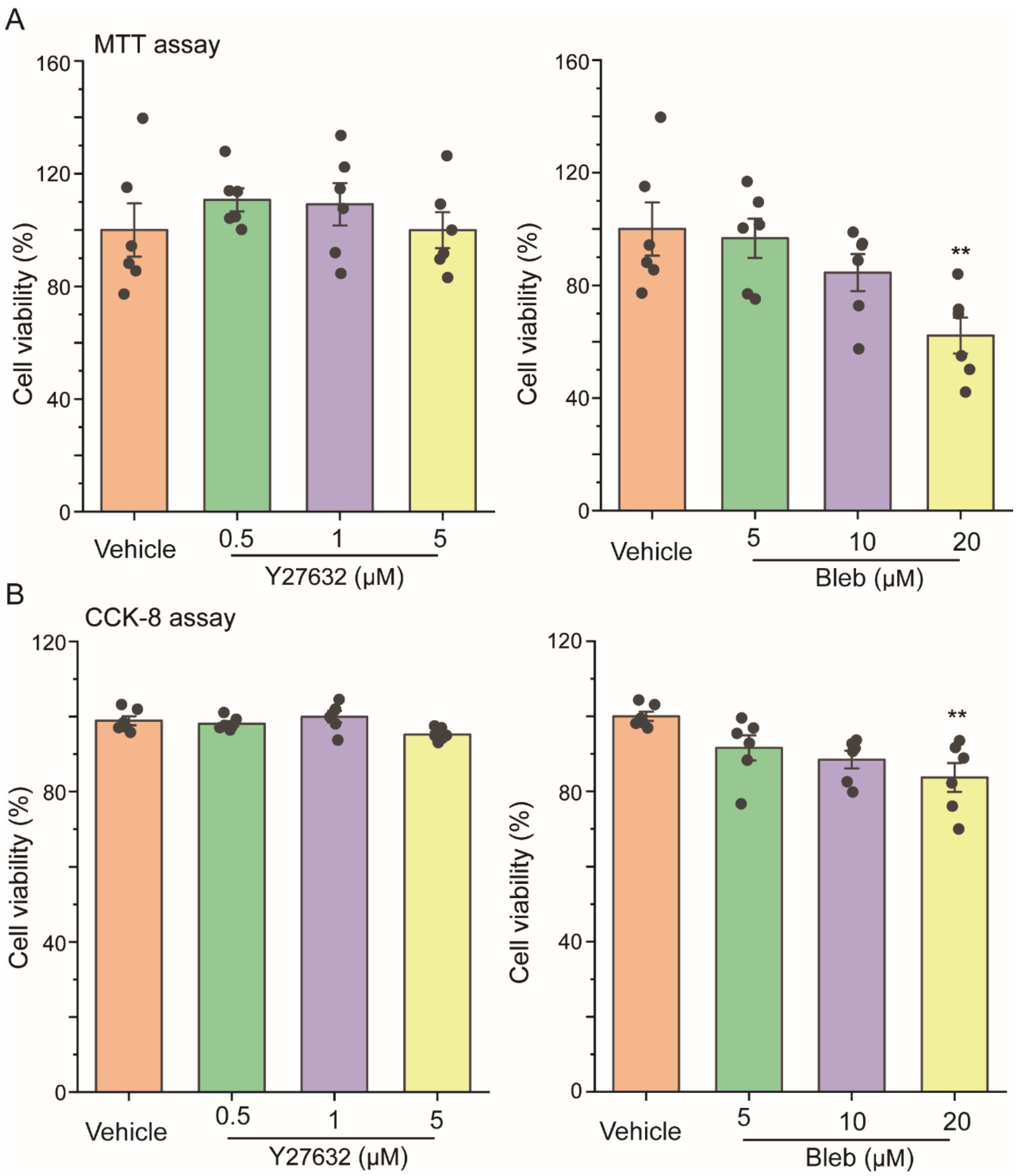

Figure S3: The effect of ROCK signaling inhibitor Y27632 (0.5, 1, and 5 μM, dissolved with DMSO) and myosin II inhibitor blebbistatin (Bleb, 5, 10, and 20 μM, dissolved with DMSO) on the cellular activity of mASMCs evaluated with MTT (**A**) and CCK-8 (**B**). n = 6. ** $P < 0.01$.

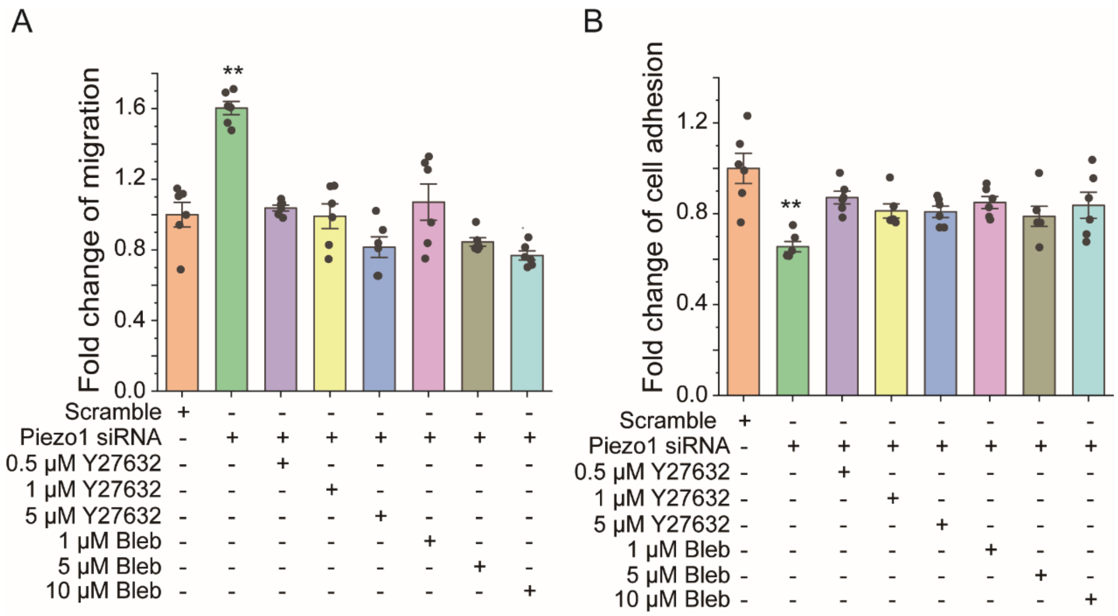

Figure S4: The effect of ROCK signaling inhibitor Y27632 (0.5, 1, and 5 µM, dissolved with DMSO) and myosin II inhibitor blebbistatin (Bleb, 1, 5, and 10 µM, dissolved with DMSO) on the loss of Piezo1-enhanced cell migration and -decreased cell adhesion evaluated with transwell assay (**A**) and cell adhesion assay (**B**). n = 6. ** $P < 0.01$.

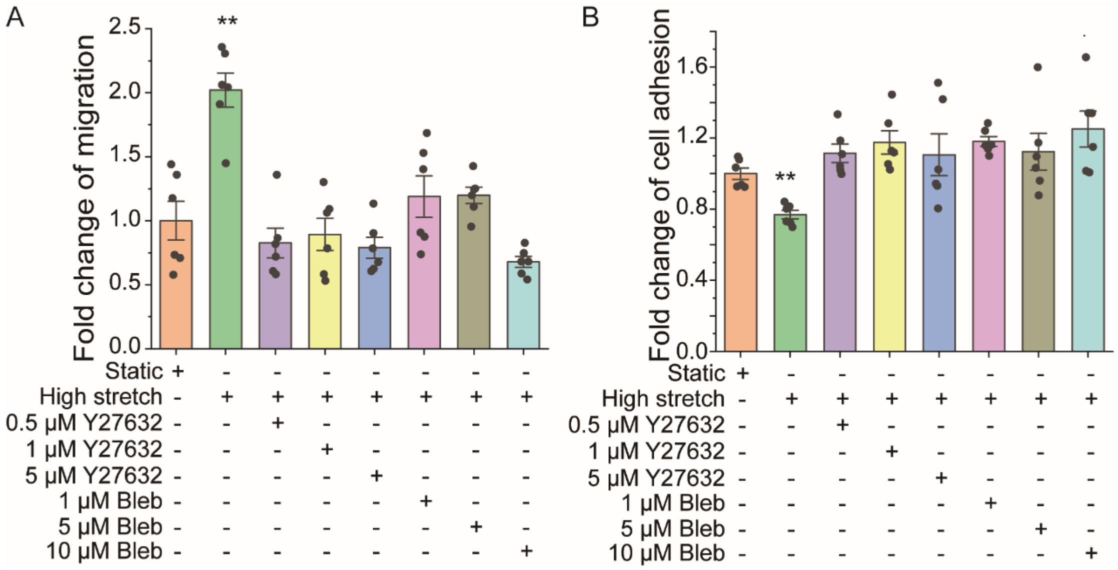

Figure S5: The effect of ROCK signaling inhibitor Y27632 (0.5, 1, and 5 µM, dissolved with DMSO) and myosin II inhibitor blebbistatin (Bleb, 1, 5, 10 µM, dissolved with DMSO) on high stretch-enhanced cell migration and -decreased cell adhesion evaluated with transwell assay (**A**) and cell adhesion assay (**B**). n = 6. ** $P < 0.01$.

Table S1: Primer sequence of mRNA for mouse airway smooth muscle cells

No.	Genes	Forward primer	Reverse primer
1	GADPH	AGGTCGGTGTGAACGGATT TG	GGGGTCGTTGATGGCAACA
2	Integrin αV	CGGGTCCCGAGGGAAGTTA	TGGATGAGCATTCACATTTG AGA
3	Integrin β1	TGGTCAGCAACGCATATCT GG	GATCCACAAACCGCAACCT
4	Piezo1	TCATCATCCTTAACCACATG GTG	TGAAGACGATAGCTGTCAT CCA
7	Piezo1 siRNA	CCG GCAUCUACGUCAAAUATT	UAUUUGACGUAGAUGCCG GUG
8	Piezo1 scramble controls	UUCUCCGAACGUGUCACG UTT;	ACGUGACACGUUCGGAGA ATT
